# Case Report: Sneaky DCIS-like invasive ductal carcinoma of the breast in the setting of extensive DCIS

**DOI:** 10.3389/fmed.2025.1673998

**Published:** 2025-11-03

**Authors:** Chong Chen, Yateng Tie, Xinjuan Zhang, Niuniu Hou, Yun Song, Dong Ren

**Affiliations:** ^1^Department of Thyroid and Breast Surgery, Air Force 986(th) Hospital, Air Force Medical University, Xi'an, Shaanxi, China; ^2^Department of General Surgery, Air Force 986(th) Hospital, Air Force Medical University, Xi'an, Shaanxi, China; ^3^Department of Aerospace Medical Training, School of Aerospace Medicine, Air Force Military Medical University, Xi'an, China; ^4^Department of Pathology, The First Affiliated Hospital of Xi'an Jiaotong University, Xi'an, China; ^5^Department of Pathology and Laboratory Medicine, University of California Irvine, Orange, CA, United States

**Keywords:** ductal carcinoma *in situ*, invasive ductal carcinoma, breast cancer, breast tumor, DCIS-like IDC

## Abstract

In most cases, invasive ductal carcinoma (IDC) of the breast is identifiable when it presents with classic infiltrative growth patterns. However, a subset of IDC can present in a very sneaky way, significantly mimicking the appearance of ductal carcinoma *in situ* (DCIS). In this condition, it is much easier to miss the invasive component without pulling ancillary staining when morphologic findings are extremely compatible with DCIS, especially the diagnosis of DCIS was made on the previous biopsy. Here, we report the case of a 55-year-old female patient who was found to have microcalcifications at the 11:00 o’clock position in the right posterior breast during a routine mammographic examination. A biopsy of the calcification area performed at an outside hospital reported high-grade DCIS (ER+, PR−). Histologic examination of the subsequent mastectomy specimen at our institution showed two separate areas that closely resembled DCIS. Immunohistochemical (IHC) staining showed that all myoepithelial markers—smooth muscle myosin heavy chain (SMMHC), p63, CK5/6, and S100—were retained at the periphery of the expanded acini in one of the areas. Unexpectedly and surprisingly, myoepithelial markers were completely lost at the periphery of a subset of the DCIS-looking acini in another area, a finding that was immunohistochemically consistent with the diagnosis of invasive ductal carcinoma admixed with DCIS. Knowing that invasive ductal carcinoma of the breast can exhibit a DCIS-like morphology, especially in cases where a prior biopsy has already established a diagnosis of DCIS, will enhance the awareness of pathologists to recognize invasive ductal carcinoma that mimics DCIS. In turn, this will prevent misdiagnosis and undertreatment of patients with invasive ductal carcinoma of the breast.

## 1 Introduction

Breast carcinoma is the most commonly observed malignancy in female individuals across all age groups, with risk increasing significantly with age (1). Invasive ductal carcinoma (IDC) is the most common histologic subtype of breast cancer (BC), accounting for approximately 90% of all BC cases (2). Ductal carcinoma *in situ* (DCIS) is regarded as a direct precursor to IDC and is characterized by the malignant proliferation of ductal epithelial cells within the ductal-lobular system, without evidence of stromal invasion (3). DCIS accounts for approximately 20% of newly diagnosed breast cancer cases, where approximately 25–60% of untreated DCIS cases have been reported to progress to IDC after a median follow-up of 9–24 years (4, 5).

## 2 Materials and methods

A total mastectomy was performed for this patient. The surgical resection was completed at 9:24 a.m., and the specimen was placed in 10% neutral buffered formalin at 10:24 a.m. The total fixation time from resection to submission for histologic processing was 13.5 h. Immunohistochemical (IHC) staining for SMMHC, p63, CK5/6, and S100 was performed and independently evaluated by two pathologists.

## 3 Case presentation

A 55-year-old female patient was found to have microcalcifications at the 11:00 o’clock position in the right posterior breast during a routine mammographic examination at an outside hospital. Biopsy of the calcification area showed high-grade DCIS, ER+, and PR− (pathologic slides were not available for review). The patient was transferred to our institution for mastectomy resection.

**Figure 1:**
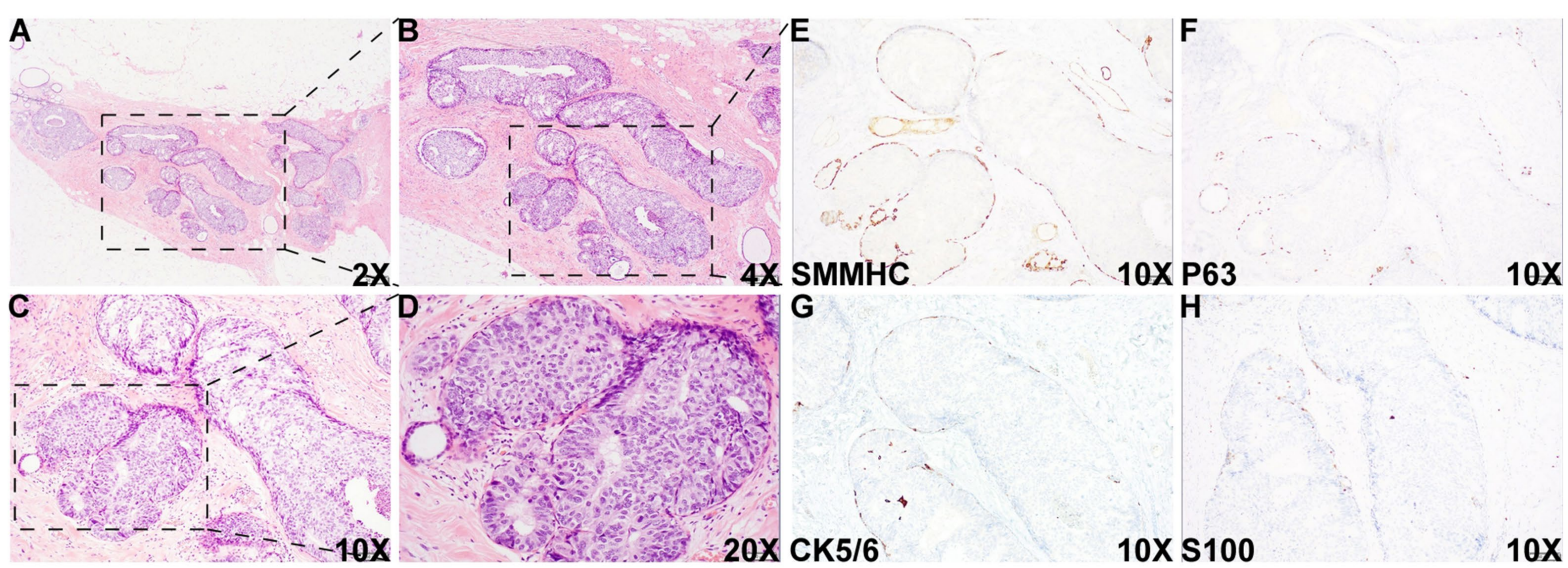
Histologic images of the DCIS area. Histologic examination of the specimen showed an area with multiple densely packed, well-bordered, expanded acini composed of monotonous epithelioid cells [2X **(A)**, 4X **(B)**, 10X **(C)**, and 20X **(D)**]. Immunohistochemical (IHC) staining showed that myoepithelial markers, such as smooth muscle myosin heavy chain (SMMHC) **(E)**, p63 **(F)**, CK5/6 **(G)**, and S100 **(H)**, were retained at the periphery of all the expanded acini, supporting the diagnosis of DCIS in this area.

**Figure 2:**
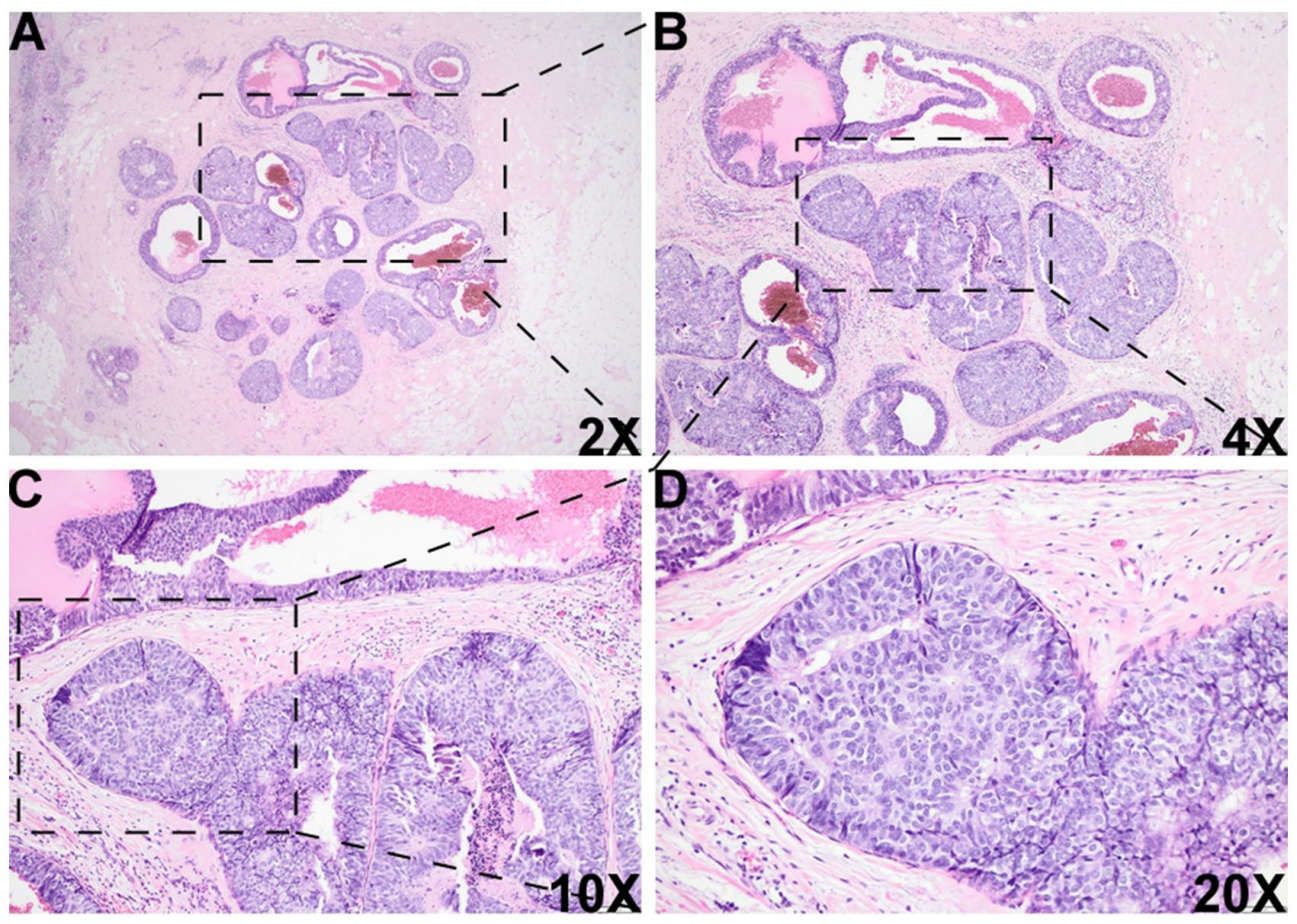
Histologic images of the DCIS-like IDC area. There was another morphologically similar area adjacent to the DCIS area, which was composed of multiple well-bordered, expanded acini with monotonous epithelioid cells and dilated cystic changes [2X **(A)**, 4X **(B)**, 10X **(C)**, and 20X **(D)**].

**Figure 3:**
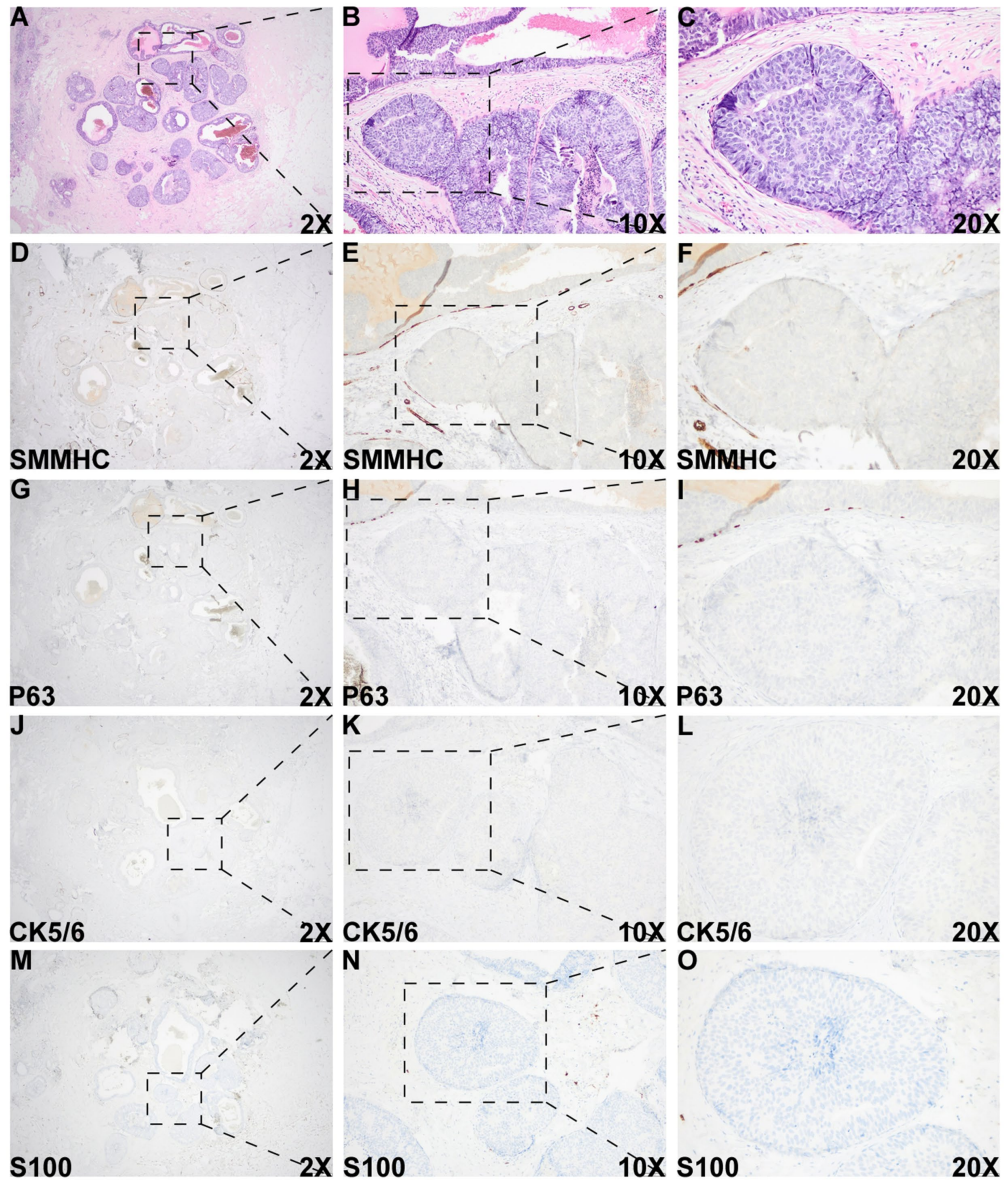
Histologic and immunohistochemical evaluation of the DCIS-like IDC area. **(A–C)** Morphology of the DCIS-like IDC area. **(D–O)** IHC staining of SMMHC **(D–F)**, p63 **(G–I)**, CK5/6 **(J–L)**, and S100 **(M–O)** in the DCIS-like IDC area. IHC staining for both the DCIS-like IDC area and the adjacent DCIS area was performed under identical conditions.

Histologic evaluation of the resection specimen revealed two separate clusters of densely packed, well-circumscribed acini composed of monotonous epithelioid cells (Figures 1A–D, 2). Immunohistochemical (IHC) staining of the first cluster (Figure 1) demonstrated retention of myoepithelial markers—smooth muscle myosin heavy chain (SMMHC; Figure 1E), p63 (Figure 1F), CK5/6 (Figure 1G), and S100 (Figure 1H)—at the periphery of all expanded acini, supporting a diagnosis of DCIS. The predominant architectural patterns were comedo and cribriform, with a nuclear grade of III. In contrast, the second tumor cluster (Figures 2, 3) showed complete loss of SMMHC, p63, CK5/6, and S100 expression at the periphery of a subset of acini morphologically resembling DCIS. Notably, SMMHC and p63 staining were retained at the periphery of DCIS acini adjacent to DCIS-like IDC acini (Figures 3B–C for SMMHC and Figures 3E–F for p63), whereas CK5/6 and S100 staining were lost in the same regions (Figures 3H,I,K,L). However, CK5/6 and S100 expression were preserved in other DCIS areas of the same tumor cluster (Supplementary Figure 1).

Overall, these IHC findings support the diagnosis of IDC admixed with DCIS. The IDC component was graded as II (6/9). As the “edge effect” or retraction artifact—where tissue separation from the stroma creates artificial periductal clear spaces—can mimic the loss of myoepithelial markers, careful distinction between true myoepithelial loss and artifact is essential. High-power magnification of the DCIS-like IDC region showed an intact stromal framework surrounding the tumor nests (Supplementary Figure 2), confirming the diagnosis of DCIS-like IDC arising in a background of DCIS.

## 4 Discussion

IDC shows significant overlap with DCIS in several aspects, including epidemiological risk factors such as age and family history, genetic factors such as BRCA1/BRCA2, and molecular markers such as ER, PR, and HER2 (6). IDC can be divided into multiple subtypes, including tubular, mucinous, papillary, cribriform, pleomorphic, and solid (6), many of which share significant histomorphological overlap with DCIS, particularly in patterns such as papillary, micropapillary, cribriform, and comedo-necrosis (7). Among these, the solid pattern of IDC is one of the most common subtypes that is often underestimated and misdiagnosed as DCIS. Several studies have reported that a DCIS-like pattern can present a diagnostic dilemma, particularly in cases where IDC is misdiagnosed as DCIS (8–10). However, most published cases describe a distinct DCIS-like IDC area. In contrast, our case involved DCIS-like IDC components that were intimately mixed with actual DCIS areas, posing a significant challenge in diagnosing IDC against a background of extensive DCIS. The aim of this case report was to highlight a major pitfall in diagnosing IDC in the context of extensive DCIS.

IDC subtypes play an important role in guiding treatment strategies and predicting prognosis in clinical practice. A study by Wang et al. demonstrated that the invasive papillary carcinoma subtype, which closely resembles DCIS, typically exhibits indolent biological behavior, low lymph node metastasis rates, and a favorable prognosis, supporting the need to avoid overtreatment (11). In contrast, the micropapillary subtype of IDC is associated with worse overall survival compared to IDC of no special type (NST); during the first 5 years, the overall survival rate was 86.2% for the micropapillary subtype versus 90.8% for IDC-NST (*p* < 0.05) (12). In this case, no recurrence or metastasis was identified on imaging or pathology during the 2-year follow-up period. These findings underscore the importance of accurate IDC subtyping, which is essential for optimizing clinical management and prognostic stratification in breast cancer patients.

A DCIS-like pattern of invasive carcinoma, characterized by well-circumscribed solid tumor nests or nodules, represents a rare pattern of invasion that can be easily mistaken for an *in situ* proliferation (10, 13, 14). As this subtype of invasive carcinoma closely resembles DCIS, confirmation of its invasive nature relies primarily on the loss of myoepithelial markers. A common diagnostic pitfall is the “edge effect” or retraction artifact, in which tissue separation from the surrounding stroma at the periphery of ducts or acini creates artificial clear spaces that may mimic the loss of myoepithelial cells. In this case, high-power magnification of H&E and IHC sections at the critical interfaces demonstrated an intact stromal framework and a true absence of myoepithelial cells within the suspicious area. Furthermore, IHC staining for multiple myoepithelial markers—including SMMHC, p63, CK5/6, and S100—was performed on both the invasive and adjacent DCIS regions within the same batch of sections. All myoepithelial markers were absent in the DCIS-like invasive area but retained in the DCIS area, confirming the diagnosis of IDC admixed with DCIS.

## 5 Conclusion

In this case, the diagnosis of DCIS was easily made without awareness of the DCIS-like IDC morphology. This case will definitely raise recognition and awareness among pathologists to always consider the possibility of IDC in expansile DCIS-like areas.

## Data Availability

The original contributions presented in the study are included in the article/Supplementary material, further inquiries can be directed to the corresponding author/s.

## References

[1] Giaquinto AN Sung H Miller KD Kramer JL Newman LA Minihan A. Breast cancer statistics, 2022. CA Cancer J Clin. (2022) 72:524–41. doi: 10.3322/caac.21754, PMID: 36190501

[2] Li CI Anderson BO Daling JR Moe RE. Trends in incidence rates of invasive lobular and ductal breast carcinoma. JAMA. (2003) 289:1421–4. doi: 10.1001/jama.289.11.1421, PMID: 12636465

[3] Badve SS Gokmen-Polar Y. Ductal carcinoma in situ of breast: update 2019. Pathology. (2019) 51:563–9. doi: 10.1016/j.pathol.2019.07.005, PMID: 31472981 PMC6788802

[4] Sanders ME Schuyler PA Dupont WD Page DL. The natural history of low-grade ductal carcinoma in situ of the breast in women treated by biopsy only revealed over 30 years of long-term follow-up. Cancer. (2005) 103:2481–4. doi: 10.1002/cncr.21069, PMID: 15884091

[5] Collins LC Tamimi RM Baer HJ Connolly JL Colditz GA Schnitt SJ. Outcome of patients with ductal carcinoma in situ untreated after diagnostic biopsy: results from the nurses' health study. Cancer. (2005) 103:1778–84. doi: 10.1002/cncr.20979, PMID: 15770688

[6] Harvey JA. Unusual breast cancers: useful clues to expanding the differential diagnosis. Radiology. (2007) 242:683–94. doi: 10.1148/radiol.2423051631, PMID: 17325062

[7] Shaaban AM Hilton B Clements K Provenzano E Cheung S Wallis MG. Pathological features of 11,337 patients with primary ductal carcinoma in situ (DCIS) and subsequent events: results from the UK sloane project. Br J Cancer. (2021) 124:1009–17. doi: 10.1038/s41416-020-01152-5, PMID: 33199800 PMC7921398

[8] Kordek R. Ductal carcinoma in situ-like structures in metastatic breast carcinoma. Pathol Res Pract. (2005) 200:831–4. doi: 10.1016/j.prp.2004.08.006, PMID: 15792128

[9] Zhang RR Man YG Vang R Saenger JS Barner R Wheeler DT. A subset of morphologically distinct mammary myoepithelial cells lacks corresponding immunophenotypic markers. Breast Cancer Res. (2003) 5:R151–6. doi: 10.1186/bcr635, PMID: 12927046 PMC314436

[10] Mohan N Black JO Schwartz MR Zhai QJ. Invasive ductal carcinoma with in situ pattern: how to avoid this diagnostic pitfall? Am J Transl Res. (2016) 8:3337–41. PMID: 27648124 PMC5009386

[11] Wang S Zhang Q Mao X. Invasive papillary carcinoma of the breast. Front Oncol. (2024) 14:1374091. doi: 10.3389/fonc.2024.1374091, PMID: 38601769 PMC11004302

[12] Aker FV Ekren E Dogan M Gurleyik G Tanrikulu E Oven BB. Clinicopathological features and prognosis of invasive micropapillary carcinoma compared to invasive ductal carcinoma-NOS: worse or better? J Coll Physicians Surg Pak. (2022) 32:1196–201. doi: 10.29271/jcpsp.2022.09.1196, PMID: 36089720

[13] Coyne J. Metastatic mammary carcinoma with DCIS-like morphology: a report of two cases. Int J Surg Pathol. (2012) 20:485–7. doi: 10.1177/1066896911435724, PMID: 22297833

[14] Pervez S Khan H. Infiltrating ductal carcinoma breast with central necrosis closely mimicking ductal carcinoma in situ (comedo type): a case series. J Med Case Rep. (2007) 1:83. doi: 10.1186/1752-1947-1-83, PMID: 17825107 PMC2014768

