# Supplementary figures and images for: Case Report: Sneaky DCIS-like invasive ductal carcinoma of the breast in the setting of extensive DCIS
Source: Front Med (Lausanne). 2025 Nov 3;12:1673998. doi: 10.3389/fmed.2025.1673998 (PMC12622304; doi:10.3389/fmed.2025.1673998)

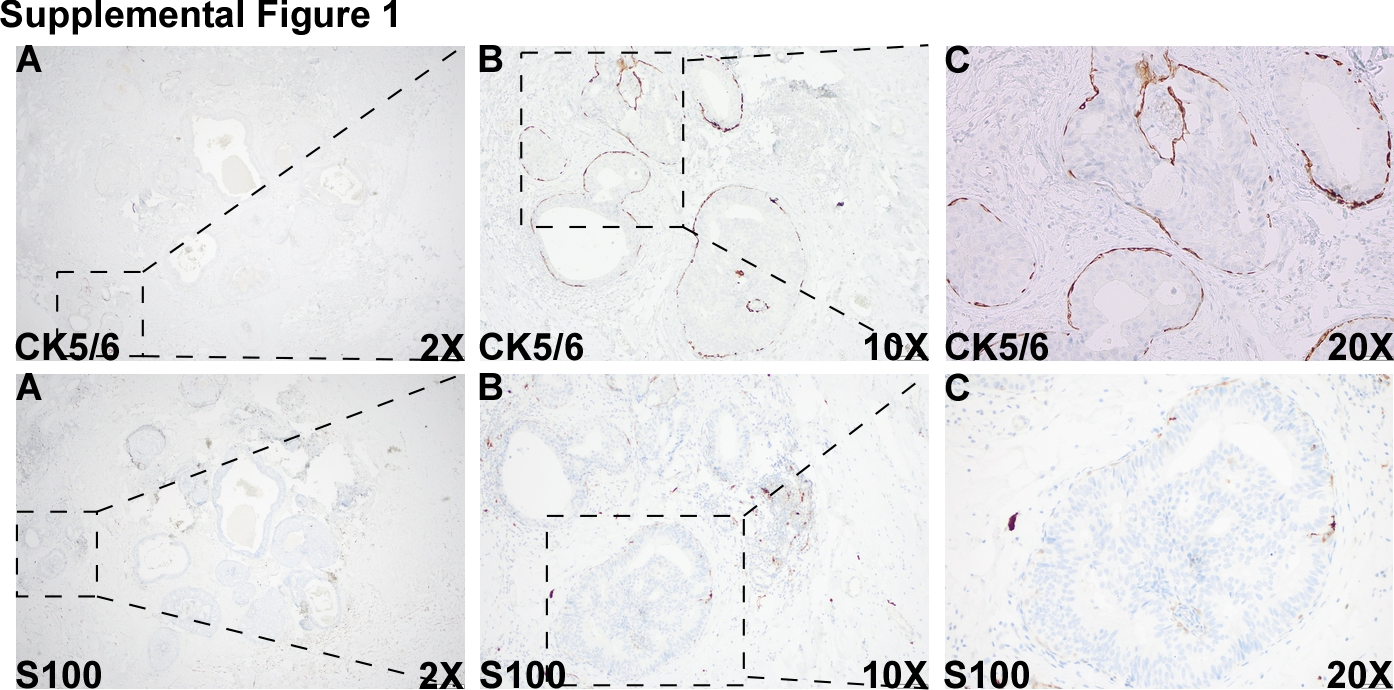

Supplement: Supplementary file 1 [file Image_1.JPEG]

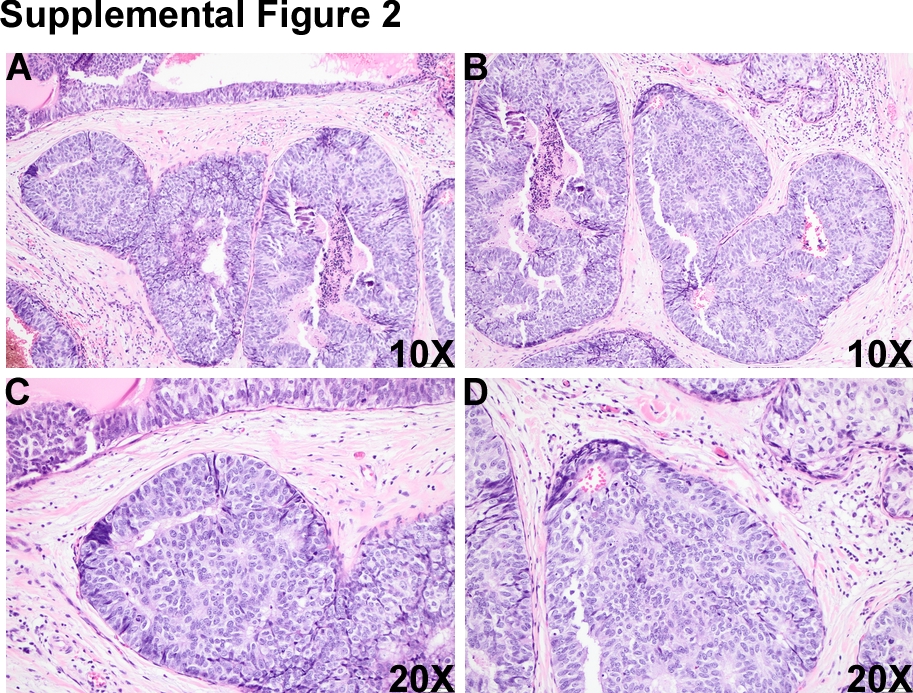

Supplement: Supplementary file 2 [file Image_2.JPEG]
